# Extending the elderly- and risk-group programme of vaccination against seasonal influenza in England and Wales: a cost-effectiveness study

**DOI:** 10.1186/s12916-015-0452-y

**Published:** 2015-10-13

**Authors:** Marc Baguelin, Anton Camacho, Stefan Flasche, W. John Edmunds

**Affiliations:** Respiratory Diseases Department, Public Health England, London, UK; Centre for the Mathematical Modelling of Infectious Diseases, Department of Infectious Disease Epidemiology, London School of Hygiene and Tropical Medicine, Keppel Street, London, UK

**Keywords:** Children, Cost-effectiveness, Influenza, Respiratory infections, Vaccination

## Abstract

**Background:**

The present study aims to evaluate the cost-effectiveness of extending the pre-2013 influenza immunisation programme for high-risk and elderly individuals to those at low risk of developing complications following infection with seasonal influenza.

**Methods:**

We performed an economic evaluation comparing different extensions of the pre-2013 influenza programme to seven possible age groups of low-risk individuals (aged 2–4 years, 50–64 years, 5–16 years, 2–4 and 50–64 years, 2–16 years, 2–16 and 50–64 years, and 2–64 years). These extensions are evaluated incrementally on four base scenarios (no vaccination, risk group only with coverage as observed between 1995 and 2009, risk group and 65+, and risk group with 75 % coverage and 65+). Impact of vaccination is assessed using a transmission model built and parameterised from a previously published study. The study population is all individuals of all ages in England and Wales representing an average total of 52.6 million people over 14 influenza seasons (1995–2009).

**Results:**

The influenza programme (risk group and elderly) prior to 2013 is likely to be cost effective (incremental cost effectiveness ratio: 7,475 £/QALY, net benefit: 253 M£ [15–829]). Extension to any one of the low-risk target groups defined earlier is likely to be cost-effective. However, strategies that do not include vaccination of school-aged children are less likely to be cost-effective. The most efficient strategy is extension to the 5–16 year age group while universal vaccination (extension to all low-risk individuals over 2 years) will achieve the highest net benefit. While extension to the 2–16 year age group is likely to be very cost effective, the cost-effectiveness of extensions beyond 2–16 years is very uncertain. Extension to the 5–16 year age group would likely remain cost-effective even without herd immunity effects to other age groups. As our study includes a strong historical component, our results depend on the efficacy of the influenza vaccine remaining at levels similar to the ones achieved in the past over a long-period of time (assumed to vary between 28 % and 70 % depending of the circulating strains and age groups).

**Conclusions:**

Making use of surveillance data from over a decade in conjunction with a dynamic model, we find that vaccination of children in the United Kingdom is likely to be highly cost-effective, not only for their own benefit but also to reduce the disease burden in the rest of the community.

## Background

Most high income countries recommend that influenza vaccination should be targeted at individuals at highest risk of complications following influenza infection, such as those with chronic heart or lung disease, metabolic or renal disease, or immunodeficiencies [1], as well as the elderly [2]. Recently, many countries have expanded their definition of high risk to also include pregnant women and the obese, and in 2012 the World Health Organization (WHO) also recommended vaccination of young children (particular those under 2 years of age) as these also have a high risk of hospitalisation given infection [3]. However, these recommendations tend to consider only the direct benefits of immunisation in individuals (preventing serious complications). Widespread vaccination might also impact on those who are not directly reached by the programme, particularly if vaccination is aimed at children, who are known to play a key role in the spread of influenza [4–6]. Allocating limited health resources to exploit indirect protection could prove to be a more cost-effective alternative than targeting only those at highest risk of disease but with little role in the transmission of influenza [7].

Estimating the effectiveness and cost-effectiveness of transmission-reducing influenza vaccination strategies is complicated as it requires either large-scale community randomised trials [8] or a mathematical model capable of accurately simulating influenza transmission. Both approaches also need to run over many years as influenza seasons vary in severity due to differences in the virus subtype and intensity of circulation.

Recently, we have reconstructed the transmission dynamics of influenza A H3N2, H1N1, and influenza B in the United Kingdom (UK) from 1995 to 2009 [9]. This allowed us to test alternative vaccination scenarios and evaluate their population impact over a large time frame. This mathematical model formed the first part of a series of three linked studies presented to the Joint Committee on Vaccination and Immunisation in the UK. These studies prompted the Joint Committee on Vaccination and Immunisation to recommend, in the summer of 2012, to extend the influenza immunisation programme in the UK to all children aged 2–17 years [10]. The second of these three studies, estimating the burden of influenza, has been recently published [11]. We present here the last of these studies, which assimilates the information from the earlier two aspects (modelling and burden estimates) to look at the cost-effectiveness of extending the seasonal influenza immunisation programme.

In particular, we explore whether vaccination of high-risk individuals and the elderly (the strategy in place in the UK since 2000) should be supplemented by vaccination of low-risk groups of different ages (from young children to all 50–64 year olds). In so doing we assess whether vaccination of transmitters (low-risk school aged children) is a more efficient use of limited resources than targeting groups with the highest disease burden among those not yet targeted by the elderly- and risk-group strategy (younger children and adults aged 50–64 years). Furthermore, in addition to the original study and in order to generalise our findings to other settings, we employed this framework to explore the cost effectiveness of extending the programme to low-risk groups under different counterfactual scenarios – for instance, without a programme targeted at high-risk individuals or the elderly.

## Methods

We performed a cost-effectiveness analysis of alternative influenza vaccination policies from the perspective of the health service and personal social services, as recommended in the UK [12].

### Vaccination programmes evaluated

We evaluated the impact of the seasonal influenza vaccination under seven possible scenarios of extension to low-risk individuals in the following age groups (ordered by size: 2–4 years, 50–64 years, 5–16 years, 2–4 and 50–64 years, 2–16 years, 2–16 and 50–64 years, and 2–64 years) incremental on four different base policies (no vaccination, risk groups only, risk groups and elderly, and risk groups and elderly with risk group at 75 % coverage). The base case of our study is the ‘risk group and elderly’ policy which was recommended in the UK from 2000 to the time of this analysis (i.e. 2012). The coverage of the extended programme is assumed to reach 50 % while coverage of the base programme is assumed to remain the same as the actual coverage observed in the different years of the study. The detailed figures of the coverage for the different age- and risk-groups for the different years of the study were presented in Table S1 of Baguelin et al. [9]. Coverage remained modest in low-risk groups but increased steadily in risk groups during the early years of the study following efforts to promote vaccination for individuals at high risk of complications. In the late years of the study, risk group vaccination plateaued around 20 % in children (aged 1–14 years) and just below 50 % in adults. Levels of vaccination in the elderly were comparatively high, remaining constantly between 70–75 % starting from the 2003/2004 season. In the base case, vaccination is assumed to be 70 % in the elderly for the year before the 2000/2001 season when the change of policy was decided. In the risk group-only base policy, the coverage in the low-risk elderly is assumed to remain at 29.3 % after 2000, the average coverage in the previous years in that group.

As there is significant uncertainty in the coverage that may be achieved for the extensions, the base-case assumption of 50 % was varied between 15 %, 30 %, and 70 %. The costs of expanding coverage in the high-risk individuals were assumed to increase linearly with coverage (constant marginal returns). The 53 scenarios evaluated are listed in Table 1 with each cell of the table representing one scenario.

**Table 1:** Vaccine strategies evaluated in the study

		Base policy						
		No vaccination	Risk group only (Pre-2000 )	Risk group and elderly (Post-2000)				Risk group (75 %) and elderly
Extension	None	X	X	X				X
	2–4 years	50 %	50 %	15 %	30 %	50 %	70 %	50 %
	50–64 years	50 %	50 %	15 %	30 %	50 %	70 %	50 %
	5–16 years	50 %	50 %	15 %	30 %	50 %	70 %	50 %
	2–4 and 50–64 years	50 %	50 %	15 %	30 %	50 %	70 %	50 %
	2–16 years	50 %	50 %	15 %	30 %	50 %	70 %	50 %
	2–16 and 50–64 years	50 %	50 %	15 %	30 %	50 %	70 %	50 %
	2–64 years	50 %	50 %	15 %	30 %	50 %	70 %	50 %

Vaccination strategies evaluated in this study. Strategies are built on a base policy and an extension to groups among the population at low risk of complications. Coverage figures are for the extension while for the base policy historical figures observed other the length of the study are used. A total of 53 scenarios have been evaluated, each of them represented by a cell in this table

### Model

We utilise the reconstructed epidemic profiles from Baguelin et al. [9] and estimate the number of infections that occurred for each influenza strain by age and risk group during the 14-year study period (1995/1996 to 2008/2009) under the actual vaccination programme. In addition, we use the same model to estimate the number of infections that would have occurred under other vaccination programmes; i.e. if the vaccination programme had been extended to other (low-risk) age groups. We repeated this for the other three base programmes (no vaccination, risk group only, risk group and elderly but with a 75 % coverage in risk group).

Details of the evidence synthesis and parameter assumptions for the reconstruction of the past epidemics and the creation of alternative vaccination scenarios have been described earlier [9]. In brief, the model generates a sample of epidemiological scenarios for each type of circulating influenza strain (H1N1, H3N2, and influenza B) and each of the years of the study by using an adaptive Markov chain Monte Carlo method. This gives a set of epidemics, based on the posterior distributions of the parameters, that reproduces annual strain-specific epidemiological patterns between 1995 and 2009. For each of these epidemics, alternative vaccine scenarios are generated by running the same model with a different vaccine calendar for that year and scenario. This then generates the estimated number of infections that would have occurred under alternative strategies.

We assume in the base case that vaccine coverage is 50 % among low-risk groups and that individuals are immunised in the autumn. Parameters and assumptions of the transmission model can be found in more details in Baguelin et al. [9].

### Linking estimates of infection risks with disease burden risks

In addition to the number of infections associated with each scenario, the number of associated health outcomes, such as number of clinical cases, general practitioner (GP) consultations, hospitalisations, and deaths, are estimated. The proportion of clinical cases among infections is obtained from Carrat et al. [13] (Table 2). To link the model-derived number of infections to the other health outcomes (GP consultations, hospitalisations, and deaths), we use an age-specific (and strain and risk specific when available) negative binomial regression model with identity link and intercept. These health outcomes are taken from Cromer et al. [11] and regressed in the year they are available using the model estimates of infection during these years as predictors. This is similar to the estimation process of the number of deaths resulting from different vaccine scenarios through linking of model-derived infection estimates with death estimates via regression, which has been previously described [9]. A major difference between the study by Baguelin et al. [9] and the present study however, is that the deaths results used herein were obtained from the study by Cromer et al. [11], where deaths are defined as deaths following hospitalisations. This provides a more conservative estimate than regressing against, for example, all-cause mortality. To account for uncertainty in the estimated number of the different health outcomes (including deaths) attributable to influenza per year, we have sampled the estimates from Cromer et al. [11] using the resulting normal distributions from the regression study rather the mean estimates. Additionally, the GP consultation data were not available by risk group status. The fraction of GP consultations for influenza-like illness (ILI) attributed by risk group is based on the risk group prevalence and the relative risk of an ILI consultation in those in any risk group compared to the low-risk population in an internet-based cohort [14, 15].

**Table 2:** Parameters of the economic model

Parameter	Estimate	Uncertainty	Source
Relative risk of consulting a GP in a risk group	1.51	Normal (μ = 1.51, sd = 0.18)	Flusurvey (http://flusurvey.org.uk/) cohort [14]
Cost of vaccination	15.85	Triangular (vertices 12, 15.55, 20)	Personal communication (Department of Health)
Febrile cases	0.406	Triangular on [0.309–0.513]	Review of volunteer studies [13]
All ARI cases	0.645	Triangular on [0.546–0.733]	Review of volunteer studies [13]
QALY loss per non-fatal ILI case	7.49 × 10^-3^	Bootstrap from data on H1N1 pdm	Van Hoek et al. [21]
QALY loss per non-fatal ARI case	1.01 × 10^-3^	Normal (μ = 1.01 × 10^-3^, sd = 8.35 × 10^-5^)	Camacho et al. [14]
QALY loss per hospitalisation	0.018	Normal (mu=0.018, sigma=0.0018)	Siddiqui et al. [22]
Hospital cost (per episode)	£840	Lognormal (normal μ = 839, normal σ = 192.1)	Baguelin et al. [7]
GP cost (per consultation)	£37	Lognormal (normal μ = 37, normal σ = 8.4)	Baguelin et al. [7]

Values of parameters used in the economic model and their associated uncertainty. As part of the probabilistic sensitivity analysis, more uncertainty is added by using the distributions of estimates from Cromer et al. [11] rather than the mean estimates when estimating the risk of different health outcomes following one influenza infection

GP, General practitioner; ARI, Acute (non-influenza-like) respiratory infections; ILI, Influenza-like illness; QALY, Quality-adjusted life year

### Efficacy and cost of vaccines

A recent Cochrane review [16] suggested that vaccine efficacy was 73 % in years in which the vaccine was well-matched, and 44 % in years when there was a poor match between the vaccine and circulating strains. In addition, a recent analysis by Fleming et al. [17] on seasonal influenza vaccine efficacy, suggested that efficacy was lower in the elderly (46 %) compared to younger adults (70 %). Since all of the studies included in the Cochrane review were performed on healthy young adults, we assumed that efficacy was 70 % and 46 % in those under or over 65 years of age, respectively, in a well-matched year, which was reduced to 42 % and 28 % in poorly matching years. We assume that children would be immunized with a live attenuated influenza vaccine and that this type of vaccine will produce similar protection to the current trivalent inactivated vaccine in adults [18].

In the base-case analysis, we also assume that the live attenuated influenza vaccine (LAIV) used for children from 2–16 years of age, has a cost per dose similar to the trivalent inactivated vaccine (Table 2). Costs of the vaccination programme were guided by the Department of Health (Stephen Robinson, personal communication). Delivery costs were estimated to be £9.64 per dose (item of service payment of £7.64 and dispensing fee of £2 based on the middle of a range set out in Annex G of the Statement of Financial Entitlements [19, 20]). The average reimbursement rate (cost per dose of vaccine) was estimated to be £6.21, giving an overall estimate of £15.85 per dose of vaccine. This was varied between £12 and £20 per dose in the probabilistic sensitivity analysis.

### Outcome measures and cost effectiveness analysis

Estimates of the health-related quality of life (QoL) loss associated with non-fatal cases of influenza were taken from the literature [21, 22] (Table 2). Fatal cases were assumed to also lose the average age- and risk-specific discounted quality-adjusted life expectancy. The risk- and age-specific life-expectancy was estimated from a survival analysis of the Royal College of General Practitioners Weekly Returns Service data from 2005 to 2008, using the method described by Melegaro and Edmunds [23]. Age-specific QoL weights were taken from Kind et al. [24], using the EQ-5D rating scale. Data for children were absent, and it was assumed that their average QoL weight was somewhat higher (at 0.9) than in the 18–20 year age group (the youngest age group sampled). Risk group specific data were not available, and so both groups were assumed to have the same average QoL weight. In the base-case lost life-expectancy (as a result of death from influenza), was discounted to its present value at a rate of 3.5 % per annum, as recommended by the National Institute for Health and Care Excellence (NICE) [12]. This was varied as part of the sensitivity analysis.

Influenza seasons vary in severity and, because of this, programmes can present fluctuations of cost-effectiveness on a year-to-year basis; in severe years, it is more cost-effective to vaccinate against influenza than in mild years. As we are interested in the impact of the programme over several years independently of year-on-year variations, the cost per Quality-Adjusted Life Year (QALY) gained are presented as long-term outcomes, in which the impact is averaged over a period of 10 years, each of which was randomly chosen among the study years. We also assume in our model that vaccination in a particular year does not provide protection in the following years. We thus do not model potential residual protection in the face of waning antibodies and drift of circulating viruses. Consequently, the horizon of any studied policy is one season and we do not need to apply discount rates for costs.

Results are also presented in the form of net benefit (NB), where QALYs gained are monetised by assuming that the marginal willingness to pay for a QALY is £25,000. To ease interpretation, most tables and figures are presented as changes in the costs and benefits compared with the strategy in place before the decision to implement a paediatric programme (i.e. the programme in place before 2013). The policy in place in the UK prior to 2013 is similar to the WHO recommendation and will be later referred in this paper as the elderly- and risk-group programme. In addition to comparing alternative programmes to this elderly- and risk-group programme, an incremental analysis is also performed (Table 3) in which the alternatives are ranked in terms of net costs and the additional benefits and costs of the next most costly strategy is compared, having removed dominated alternatives. We performed probabilistic sensitivity analysis for the economic model using the applicable distributions for the different parameters (Table 2).

**Table 3:** Incremental cost-effectiveness ratios (ICERs)

Increment	ICER (£/QALY)	Net benefit in £M	95 % credibility interval
Elderly- and risk-group → 2–4 y	2,613	74	(12–265.7)
2–4 y → 5–16 y	1,569	384.4	(85.4–1309.6)
5–16 y → 2–16 y	3,414	58.7	(8.4–212.8)
2–16 y → 2–16 y & 50–64 y	8,093	75.9	(–11.5 to 346.1)
2–16 y & 50–64 y → 2–64 y	8,868	198.8	(–46.8 to 950.8)

Table of the incremental cost-effectiveness ratios and net benefit for the proposed immunization strategies. Strategies are ordered in terms of their net cost, and the incremental costs and incremental benefits of the next most expensive strategy is compared with the previous one. Dominated strategies (50–64 year age group and 2–4 and 50–64 year age groups) have been excluded

QALY, Quality-adjusted life year

### Sensitivity analysis

#### Net benefit derived from an extension of the programme to children

We assessed the benefit derived from an extension of the programme to children (in terms of non-fatal and death-associated QALYs gained per year and individual) in the different age- and risk-groups for the extension to 2–16-year-old, low-risk children. We derived the benefit to children using two methods.

In the first method, we used a simple calculation using a conservative estimate of the cost and QALYs gained through the direct effect of the programme for one vaccinated child. For this, we divided the cost of the vaccine (an upper boundary of cost as cost would be saved through reduced GP consultations and hospitalisations) by the average differential QALY directly gained per vaccinated child. The differential direct QALY per child was equal to the vaccine efficacy multiplied by the probability of developing clinical symptoms (clinical attack rate) and the QALY loss per febrile episode. From this, we derived a conservative minimum attack rate sufficient for the programme to be cost-effective from the direct effect alone.

In the second method, we calculated the group specific incremental cost effectiveness ratio (ICER) and NB by restricting the benefits to those gained in the 2–16 age group. We also calculated what should be the QALY loss associated with LAIV vaccination necessary to cancel the positive impact (in term of NB) in this age group.

#### Sensitivity to the cost of the vaccine

To reflect that the vaccination programme in children might result in higher cost per vaccine dose (whether because of a different delivery model or price per dose), we also performed a sensitivity analysis to vaccine costs; as an alternative to the base case we assume that the costs per dose would increase by £6 in children, bringing the overall cost per dose to be £21.85 in this age group. The choice of increasing by £6, though relatively arbitrary, reflects a plausible increase of almost 40 % compared with the adult cost per dose.

#### Sensitivity to non-influenza-like acute respiratory infections (ARIs)

In the base case, we adopted the conservative position of ignoring the QALY losses associated with acute (non-influenza-like) respiratory infections (ARI). There is little information on the QALY losses associated with ARI to attribute these episodes to influenza. In the sensitivity analysis, we included non-fatal ARI QALY losses through combining recently estimated QALY losses associated with ARI [14] and estimates of the fraction of infections that lead to non-febrile ARI [13].

## Results

### Impact and cost effectiveness of the elderly- and risk-group programme

We calculated the age-specific attack rates during the period of the study defined as the proportion in a given age group experiencing a febrile episode due to one of the three circulating strains of influenza. Results from the model show that the most affected age group in terms of clinical attack rates are children aged 5–14 (15 %) due to high contact rates and limited immunity. The 15–44 and the 6-month to 4-year-old age groups experience similar levels with 13 % and 11 % of attack rates, respectively, while the youngest and eldest age groups present smaller attack rates due to less contact and immunity from either maternal antibodies, past history of exposure to circulating viruses, or immunisation (0–6 months, 7.7 %; 45–64 years, 7.5 %; and over 65 years, 2.6 %). Table 4 summarises the results of the model in terms of disaggregated outcomes (annual numbers of cases, hospitalisations, costs, QALYs gained, etc.) for each of the strategies. From the results of the first two columns it can be seen that, compared with no vaccination, the elderly- and risk-group influenza programme is likely to be cost effective (ICER: 7475 £/QALY, NB: 253 M£ [15–829]).

**Table 4:** Summary results of model

Scenario	Elderly- and risk-group	No vaccination	2–4 y	50–64 y	5–16 y	2–4 & 50–64 y	2–16 y	2–16 & 50–64 y	2–64 y
Cases mean (000s)	5,325	6,474	4,914	4,668	2,882	4,269	2,559	1,971	322
Low	3,787	4,641	3,506	3,268	1,764	3,006	1,513	1,075	30
High	7,137	8,548	6,544	6,323	4,215	5,760	3,780	3,044	816
GP mean	603,095	721,152	539,893	541,134	313,892	480,317	264,246	207,979	37,544
Low	368,255	451,303	323,362	321,755	169,135	283,818	133,495	95,343	3,383
High	916,405	1,056,782	822,924	826,488	536,822	737,721	458,143	377,580	95,980
Hospitalisations mean	11,957	16,259	10,348	10,648	7,024	9,113	5,812	4,635	1,166
Low	6,861	9,439	5,951	5,993	3,279	5,079	2,509	1,778	59
High	19,152	26,641	16,820	17,307	12,299	14,952	10,468	8,753	3,223
Death mean	1,784	2,917	1,668	1,568	1,099	1,454	985	778	179
Low	643	1,081	602	573	346	530	289	198	6
High	3,930	6,440	3,677	3,386	2,507	3,180	2,268	1,887	625
Non death QALYs mean	40,514	49,365	37,375	35,495	21,896	32,450	19,446	14,946	2,429
Low	3,852	4,752	3,555	3,353	2,054	3,087	1,794	1,337	81
High	126,421	152,964	115,649	111,676	67,157	101,865	58,597	45,899	10,242
Death associated QALYs mean	13,554	21,660	12,684	11,932	8,371	11,073	7,508	5,936	1,316
Low	5,711	8,669	5,279	4,917	2,962	4,497	2,561	1,791	52
High	28,198	44,823	26,431	24,196	17,590	22,877	16,155	13,454	4,465
QALYs loss total mean	54,068	71,025	50,059	47,426	30,267	43,523	26,953	20,882	3,745
Low	13,253	19,358	12,387	11,767	7,954	10,915	6,940	5,336	380
High	141,995	177,528	131,318	125,105	76,379	114,248	68,559	52,870	11,601
Mean programme cost (£K)	134,817	–	149,012	187,054	191,283	201,248	205,477	257,714	418,988
Low	105,494	–	117,032	147,680	151,915	159,480	163,653	206,070	335,773
High	163,856	–	180,742	225,997	230,463	242,665	247,481	309,787	502,957
HC cost mean (£K)	32,607	40,664	28,886	29,184	17,669	25,615	14,788	11,687	2,378
Low	18,591	24,017	16,432	16,079	8,932	14,163	7,101	4,961	173
High	54,172	66,369	48,275	48,485	31,087	42,387	26,175	21,276	6,130
Cost total mean (£K)	167,425	40,664	177,898	216,238	208,952	226,863	220,265	269,401	421,366
Low	124,085	24,017	133,464	163,758	160,847	173,642	170,753	211,031	335,946
High	218,027	66,369	229,018	274,482	261,551	285,052	273,656	331,063	509,087

The average annual number of influenza-like illness (febrile) cases, GP consultations, etc. are shown along with measures of their distribution for each of the strategies (columns)

GP, General practitioner; QALY, Quality-adjusted life year

### Cost effectiveness of the possible extensions

The extensions to the elderly- and risk-group programme are presented in the seven right-hand columns of Table 4. They are ordered in terms of their net cost from left to right. Hence, vaccination of the low-risk pre-school children is the least expensive extension, followed by vaccination of the 50–64 year age group and so on. There is a general trend for the more expensive options to be more effective (reducing cases, deaths and QALYs lost more than the cheaper alternative), but this is not always the case. Vaccination of school children, for instance, results in fewer cases and deaths than vaccination of 2–4 and 50–64 year olds is estimated to, although targeting fewer individuals. Note that a large proportion of the QALYs lost (~25 %) is estimated to be due to deaths. Note also that the majority of the net costs of the programme are associated with the cost of vaccination. Savings (in terms of health care costs) are relatively small.

**Fig. 1:**
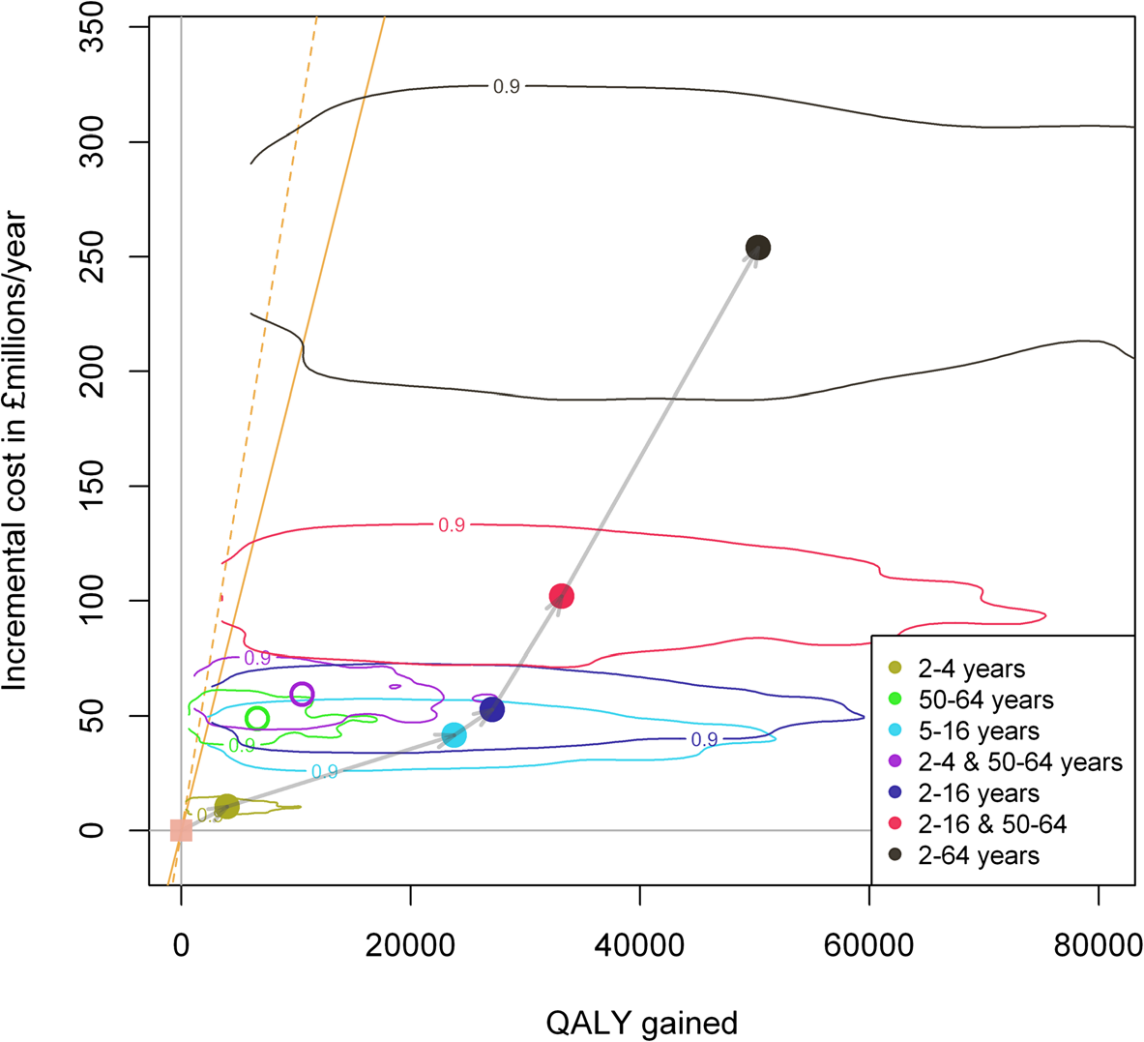
Incremental analysis with costs and quality-adjusted life years (QALYs) gained. Estimated change in costs and QALYs gained over the elderly- and risk-group strategy, for each of the extensions to the vaccination programme. Note that the comparison in each case is with the elderly- and risk-group strategy. Each contour line represents 90 % of the Monte Carlo simulations with the coloured point inside being the mean outcome of the scenario. The two diagonal lines represent £20,000 (solid) and £30,000 per QALY gained. Unfilled circles indicate strategies which are dominated by others. The arrows indicate the pathway of increasing costs for the incremental analysis

Extension of the elderly- and risk-group immunisation strategy against seasonal influenza to either one of the target groups defined earlier is likely to be cost-effective (Fig. 1). However, strategies that do not include the vaccination of school-aged children are less likely to be cost-effective. The most efficient strategy is extension to 5–16-year-old children while universal vaccination will achieve the highest NB (Table 5).

**Table 5:** Sensitivity to discount rates and coverage in risk groups

a) 3.5 % discount (Base case)							
Extension to	2–4	50–64	5–16	2–4 + 50–64	2–16	2–16 + 50–64	2–64
ICER	2613	7350	1745	5637	1949	3073	5046
Net benefit in mil £	74	91	460	164	521	604	819
Lower	12	–8	94	9	104	93	65
Higher	266	385	1577	614	1785	2107	3046
b) 1.5 % discount							
Extension to	2–4	50–64	5–16	2–4 + 50–64	2–16	2–16 + 50–64	2–64
ICER	2509	7030	1675	5398	1869	2943	4818
Net benefit	79	99	487	177	551	642	875
Lower	15	–2	115	17	126	118	108
Higher	272	394	1615	626	1820	2141	3090
c) 0 % discount							
Extension to	2–4	50–64	5–16	2–4 + 50–64	2–16	2–16 + 50–64	2–64
ICER	2406	6715	1604	5162	1788	2813	4590
Net benefit	83	107	515	190	584	680	941
Lower	18	3	135	25	148	153	159
Higher	276	401	1643	639	1864	2174	3139
d) Risk groups vaccinated with a 75 % coverage							
Extension to	2–4	50–64	5–16	2–4 + 50–64	2–16	2–16 + 50–64	2–64
ICER	2748	8677	2393	6655	2618	3844	6667
Net benefit	72	86	417	158	463	520	592
Lower	11	–17	71	–3	75	59	–17
Higher	258	381	1438	600	1607	1847	2411

Cost-effectiveness ratios (compared with the elderly- and risk-group strategy) and net benefits with associated 95 % credibility intervals using different discount rates (a–c) for future benefits (QALYs) and a different baseline (d) for the coverage in risk-groups (75 %)

ICER, Incremental cost effectiveness ratio

We estimated the incremental cost effectiveness ratios and NBs of the vaccination scenarios in the order of increasing costs of the programmes (Fig. 1 and Table 3). Dominated strategies were excluded. Gradual extension of the programme to all children is cost effective. Inclusion of children aged 5–16 years was found to be the most cost effective extension in the incremental analysis. Further extension to adults and even all individuals remains cost effective. However, it should be noted that more than 10 % of simulations fail to be cost-effective.

### Re-evaluation of historical changes

Extending the programme in the UK in 2000 from a risk-group-specific strategy to include those aged 65 years of age and over is likely to have been marginally cost-effective but with significant uncertainty (ICER: 17,023 £/QALY, NB: 12.2 M£ [–18.6 to 66]). We find that an extension to children aged 2–16 years would have been a more cost effective extension of the programme at an ICER of 2,555 £/QALY (NB: 526.4 M£ [96–1824]).

### Strategies incremental on no vaccination

In the absence of an existing programme, implementing first the routine vaccination of children is estimated to be more cost-effective than adopting a risk-based policy as implemented in the UK pre-2000; however, the risk-based policy is cost effective as well. For the vaccination of 2–16-year-old, low-risk children alone we estimate an ICER of 1,679 £/QALY (NB: 610 M£ [145–1975]) while the pre-2000 policy of targeting mainly risk groups is estimated at 6,231 £/QALY (NB: 242 M£ [28–780]). If childhood vaccination had been in place (targeting the 2–16 year age group), it would still have been cost-effective to extend the programme to include risk groups (incremental ICER of 9,215 £/QALY, NB: 160 M£ [–19 to 623]) and then to incorporate the elderly (incremental ICER of 16,482 £/QALY, NB: 5 M£ [–14 to 39]), though this last step would have provided much smaller benefits and uncertain cost effectiveness (32 % of simulations are not under a threshold of 25,000 £/QALY).

### Scenario and sensitivity analysis

#### Discount rates and baseline of coverage in risk-groups

Table 5 (a–c) presents the sensitivity of our model estimates to the assumed discount rate. In each case, extensions of the vaccination programme from the elderly- and risk-group strategy are shown (i.e. no incremental analysis). Reducing the discount rate increases the cost-effectiveness of extending vaccination, as more QALYs are gained. However, earlier conclusions from the base case remain robust to changes in discount rates. Equally, base case results are robust to the change in baseline coverage in risk groups. Even with a coverage of 75 % in risk groups conclusions regarding incremental and individual benefits of the different extensions remained unchanged, although with slightly higher ICERs and lower NBs.

We compared an extension to the low-risk 2–16-year-olds with an increase in vaccine coverage in high-risk groups to the target level of 75 % (both incremental on the elderly- and risk-group programme). The 2–16 low-risk strategy appears marginally more cost effective with an ICER of 1949 £/QALY (NB 521 M£ [104; 1785]) compared with the ICER of 2193 £/QALY (NB 200 M£ [42; 649]) associated with increasing vaccine coverage within the at risk population.

#### Sensitivity to the level of coverage in the low-risk groups

**Fig. 2:**
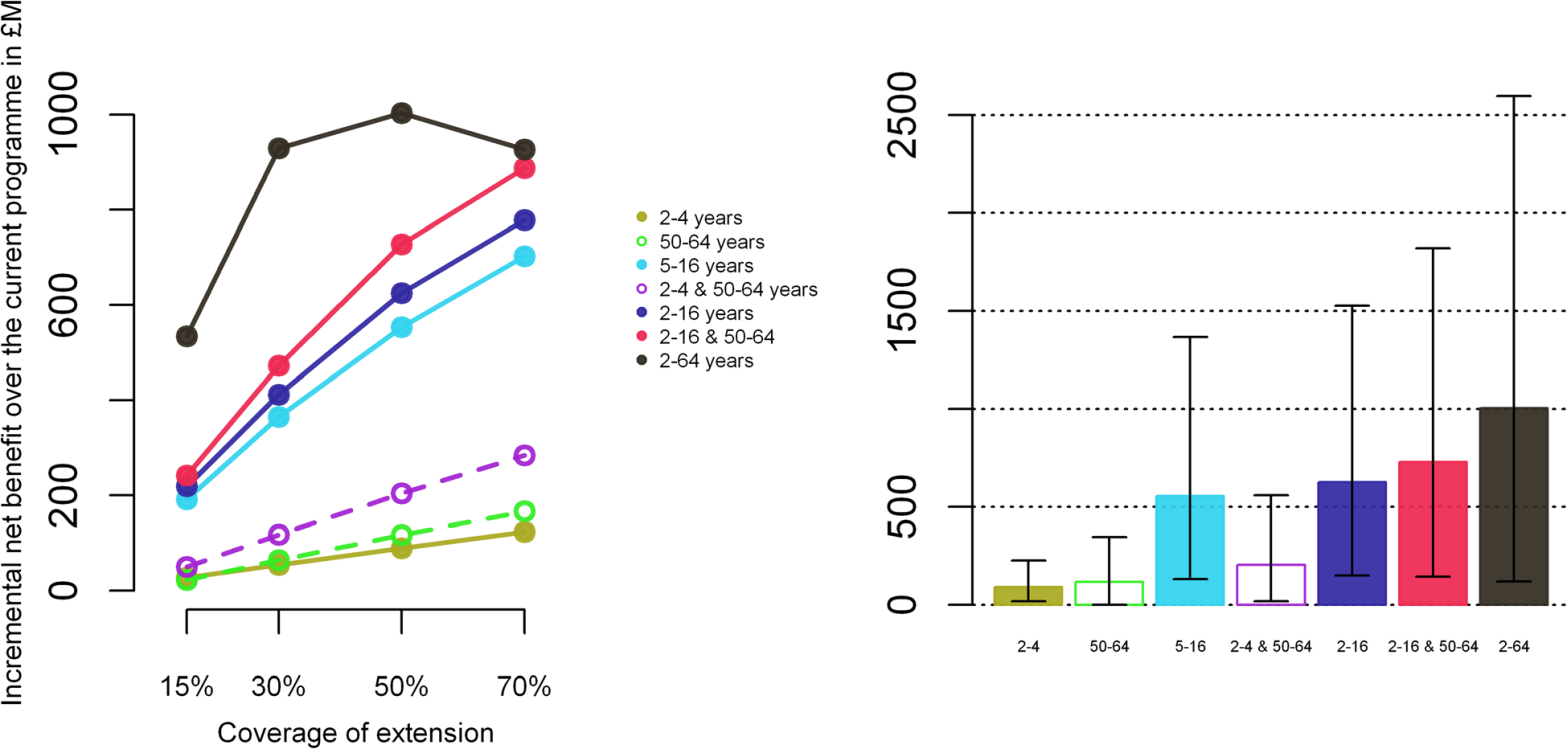
Incremental net benefit over the elderly- and risk-group programme. Incremental net benefit of different extension over the elderly- and risk-group programme for different levels of coverage (left panel) and net benefit for the base case at 50 % coverage (right panel). Dominated scenario are indicated by empty disk and bars

We estimated the NB of extending vaccination to the low-risk groups at different levels of coverage for the seven different strategies (Fig. 2). For all strategies but the universal one (extension to 2–64 years) the costs and the benefits increase with increasing coverage. However, the benefits increase at a higher rate, due to increasing herd protection in the unvaccinated and the vaccinated but not protected. For a universal vaccination programme, a turning point is reached at around 50 %, from where increasing coverage decreases the total NB (though benefits in term of QALY gained still increase).

#### Benefits derived from vaccination of children

Results from simple direct conservative calculation ignoring cost saved by reduced GP consultations and hospitalisations using cost and QALY parameters from Table 2 indicates that, for a threshold of £25,000/QALY, the vaccination programme would be cost-effective from its direct effect if more than 12 % of the child population is affected by at least one strain of influenza during the season.

**Fig. 3:**
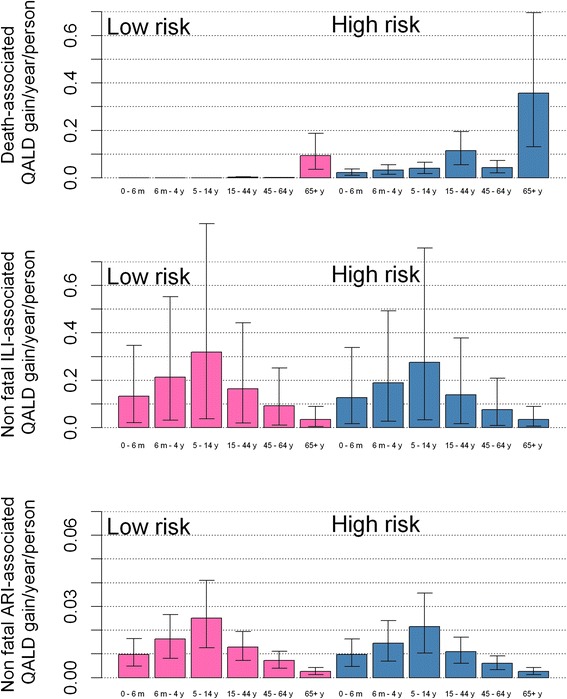
Benefits from extension to low-risk 2–16-year-old children. Benefits (in terms of non-death and death-associated Quality-Adjusted Life Days per year and per person of that age and risk group) gained from extension of vaccination to low-risk 2–16-year-old children. The benefit is given for each age and risk group (pink bars low risk, blue bars high risk). Note the change of scale in the last panel

We further studied the age, risk, and outcome-associated distribution of Quality-Adjusted Life Days (QALD) as a result of extending the programme to include low-risk children aged 2–16 years (Fig. 3). Most of the death-associated QALD gains are indirect gains in the unvaccinated population. They are concentrated in the risk groups, in particular in the elderly (65+) and younger adults (15–44) who are more likely to be in contact with children and in the low-risk elderly. The gain in non-fatal QALDs is more widely spread across the population but is highest in vaccine-targeted children aged 5–14 years and benefiting both the low-risk and the risk group of the same age (not targeted by the extension).

If one was to ignore any indirect protection, extending the elderly- and risk-group programme to children 2–16 years of age only for the benefit of these children (2–16 years), we estimate that the extension would still be cost effective. At full costs but only accounting for benefits in 2–16-year-olds, the ICER would reduce to 7,713 £/QALY and the mean NB to £81m (90 % CI –£31 to £400m). In order for the mean benefit to be offset by the QALY loss associated with LAIV vaccination (still at full costs and only accounting for benefits in the 2–16 year age group), each vaccinated child should experience a health loss of 0.39 QALD from vaccination.

#### Additional benefits due to preventing non-influenza-like ARI

In the main case, we quantify the QALY loss due to influenza infection using febrile episodes. In Fig. 3, we show the QALY loss in the different age groups associated with non-febrile acute ARI, indicating that non-fatal QALY loss is mainly associated with febrile episode though non-fatal ARI-associated QALY loss will account for an additional 10 % of non-death QALY loss.

## Discussion

We find that the strategy of vaccinating high-risk individuals and those over the age of 65 years against influenza is cost-effective. This is despite the low level of effectiveness assumed for vaccination of the elderly and the variability in impact from year to year. However, the extension of the UK programme in 2000 to include individuals over the age of 65 years, similar to current WHO recommendations, may not have made optimal use of resources. Although we find that this extension was cost effective, an alternative extension to children would have yielded a substantially higher NB.

Assessing whether the programme should be further expanded to other low-risk groups, we find that annual vaccination of children aged 5–16 years is the most cost effective option. Furthermore, vaccination of children is likely to be more cost-effective and to provide greater NB than improving coverage in the high-risk groups. Much of the impact of childhood vaccination for influenza relies on indirect protection of the community through reduced transmission. However, we find that the vaccination of children remains cost effective even if indirect effects were not taken into account. A simple conservative calculation indicates that if the attack rate in unvaccinated children is higher than 12 %, the policy is likely to be cost-effective even without taking into account indirect effects. Attack rates in excess of this level are likely in most seasons [25].

### Strength and weaknesses

This study is based on a transmission model fitted to 14 years of surveillance data and hence captures substantial amounts of inter-season variability and provides realistic parameters. It also captures the indirect impact of vaccination which allows us to distinguish between the benefits coming from direct and indirect protection. However, influenza viruses are constantly evolving and there is a chance that childhood vaccination, while retrospectively the best option, may not be optimal for future seasons if the influenza epidemiology changes substantially.

Much of the benefits derived from a childhood vaccination programme accrue to other age groups (in particular the high-risk adults). Therefore, assumptions regarding contact patterns are important. In this study, we used contemporary data on underlying social contacts [26] as input parameters into the model (priors), and further refined our estimates during the extensive model fitting procedure [9] (giving posterior distributions for these parameters). The method should therefore ensure that the resulting parameter estimates are relatively robust. Furthermore, the remaining uncertainty in these critical parameters is passed through the economic analysis. The policy choice does seem to be relatively insensitive to this (i.e. there is a relatively high probability that vaccination of children would be deemed cost-effective at £20–30,000 per QALY gained). Nevertheless, vaccination of children to prevent influenza in the population at large is controversial. A number of studies have suggested that it may be effective, although the standard of evidence has generally been weak. The essential interruption of transmission of the 2009 pandemic virus by the summer holidays in the UK [27] shows the impact that school children can have on the epidemiology of influenza in the UK, and lends support to the hypothesis that vaccination of these age groups may well bring significant benefits to others in the population. With the recent recommendation to vaccinate all children in the US, it is envisaged that further data may be forthcoming on this in the near future. It is also important to note that the benefit restricted to this age group alone is sufficient to make the child vaccination programme cost-effective even without taking into account the benefits in the other age groups.

As recommended by NICE [12], we took the perspective of the health care provider. It is possible that by restricting our attention to health-care costs, we have significantly underestimated the benefits of influenza vaccination. If a wider societal perspective had been taken (which would therefore include the costs of absenteeism, etc.), vaccinating low-risk adults would be made more cost-effective, possibly changing our conclusions about the economic attractiveness of the option.

It is possible that the analysis presented here underestimates the benefits of extending influenza vaccination to low-risk groups. The burden associated with the epidemics related to the three influenza subtypes circulating may be underestimated in this analysis [11]. For instance, the death rates were estimated from an analysis of the hospitalisation data, not all cause mortality, which would result in a far higher number of deaths being attributed to influenza, than was used herein [11]. In addition, the QoL loss associated with non-febrile cases is not included in the main analysis due to a lack of data on this [21], even though roughly one-third of all infection cases do not result in classical ILI symptoms [28]. Further, the QALY loss associated with non-hospitalised disease relies on one study only, based on confirmed cases during the 2009 influenza pandemic [21]. Though we have bootstrapped the data from this study to account for uncertainty, additional estimates resulting from infection by other strains would have allowed us to get stronger confidence in the exact loss of QALYs associated to non-fatal influenza cases and possible variation between strains. Taken together, it is likely that these results are therefore conservative. Nevertheless, the conclusions with regards to childhood vaccination are unlikely to be affected by these possible biases, since it appears that this strategy is likely to be cost-effective.

In our model, the vaccine is assumed to work by reducing transmission and vaccine efficacy is interpreted accordingly. Other modes of action of the vaccine might be consistent with observations of vaccination, such as reduction in probability or severity of symptoms. If vaccination was to reduce symptoms but not transmission, our conclusions would potentially need to be altered by considerably reducing the impact of the indirect protection provided by mass vaccination campaigns.

The model on which this study is built considers the benefits over each individual season and averages these benefits over a 14-year period. It does not consider the impact of repeated vaccinations and the associated effect on immunity in the population. Whether there will be additional benefits from protection carried forward from previous years, or a potential risk of increased susceptibility in the population due to a lack of exposure in the unvaccinated because of herd protection, is uncertain. An extension of our model framework to include immunity from natural exposure and vaccination and linking the seasons could potentially provide further insight.

### Comparison with other studies

A number of recent studies have attempted to estimate the cost-effectiveness of extending influenza vaccination over the age- and risk-group-specific policy [29–41]. These studies have either looked at extending vaccination to children [34–40], or adults aged 50–64 years [29–33], or in one case universal vaccination [41]. However, to our knowledge, no previous study has assessed the cost-effectiveness of all of these options in a single analysis. That is, no previous study has fully assessed the opportunity cost of extending vaccination by looking at alternative uses. Taken in isolation, for instance, vaccination of 50–64-year-olds may appear marginally cost-effective (see Table 3 which is broadly in line with the findings of other studies [29–33]), but when compared with vaccination of children it is dominated. A further strength is the use of a transmission dynamic framework, which is able to estimate the direct and indirect (herd-immunity) effects that may result from influenza vaccination. With very few exceptions [37, 38], model-based evaluations of influenza vaccination (reviewed in Peasah et al. [42]) uses static decision-analytic models, which ignore the effect that vaccination may have on others in the community. This leads to, for instance, the finding that vaccination of younger children is more cost-effective than school-aged children [34], as the burden of disease in younger children is greater. Our study suggests the opposite, as the increased indirect effect resulting from vaccination of school children compared to pre-schoolers exceeds the decreased direct effect. Not only did we use a transmission dynamic model, but we fitted this to a range of surveillance data using Bayesian methods [9]. Most previous economic models of influenza vaccination have relied on parameter values taken from the literature, rather than attempt to fit their models to a range of data, giving updated and refined parameter estimates and further confidence in results.

## Conclusions

Making use of surveillance data from over a decade in conjunction with a dynamic model, we find that vaccination of children in the UK is likely to be highly cost-effective, not only for their own benefit but also to reduce the disease burden in the rest of the community. The Joint Committee on Vaccination and Immunisation has recently decided to extend the influenza immunisation programme in the UK to all children aged 2–17 years [10]. The work presented here has helped shape this decision. This suggests that, in countries where children are similarly a main source for the transmission of influenza, childhood vaccination against seasonal influenza may present an opportunity to prevent a higher burden of disease at similar cost to vaccination of the elderly.

## Abbreviations

ARI: Acute (non-influenza-like) respiratory infections
GP: General practitioner
ICER: Incremental cost effectiveness ratio
ILI: Influenza-like illness
LAIV: Live attenuated influenza vaccine
NB: Net benefit
NICE: National institute for health and care excellence
QALD: Quality-adjusted life days
QALY: Quality-adjusted life year
QoL: Quality of Life
TIV: Trivalent inactivated vaccine
UK: United Kingdom
WHO: World Health Organization
